# A review of standardized high-throughput cardiovascular phenotyping with a link to metabolism in mice

**DOI:** 10.1007/s00335-023-09997-w

**Published:** 2023-06-16

**Authors:** Jiri Lindovsky, Zuzana Nichtova, Nathalia R. V. Dragano, David Pajuelo Reguera, Jan Prochazka, Helmut Fuchs, Susan Marschall, Valerie Gailus-Durner, Radislav Sedlacek, Martin Hrabě de Angelis, Jan Rozman, Nadine Spielmann

**Affiliations:** 1 Czech Centre for Phenogenomics, Institute of Molecular Genetics, Czech Academy of Sciences, Prumyslova 595, 252 50 Vestec, Czech Republic; 2 Institute of Experimental Genetics, German Mouse Clinic, Helmholtz Center Munich, German Research Center for Environmental Health, Ingolstädter Landstr. 1, 85764 Neuherberg, Germany; 3 Luxembourg Centre for Systems Biomedicine (LCSB), University of Luxembourg, Esch-sur-Alzette, Luxembourg

## Abstract

Cardiovascular diseases cause a high mortality rate worldwide and represent a major burden for health care systems. Experimental rodent models play a central role in cardiovascular disease research by effectively simulating human cardiovascular diseases. Using mice, the International Mouse Phenotyping Consortium (IMPC) aims to target each protein-coding gene and phenotype multiple organ systems in single-gene knockout models by a global network of mouse clinics. In this review, we summarize the current advances of the IMPC in cardiac research and describe in detail the diagnostic requirements of high-throughput electrocardiography and transthoracic echocardiography capable of detecting cardiac arrhythmias and cardiomyopathies in mice. Beyond that, we are linking metabolism to the heart and describing phenotypes that emerge in a set of known genes, when knocked out in mice, such as the *leptin receptor* (*Lepr*), *leptin* (*Lep*), and *Bardet–Biedl syndrome 5* (*Bbs5*). Furthermore, we are presenting not yet associated loss-of-function genes affecting both, metabolism and the cardiovascular system, such as the *RING finger protein 10* (*Rfn10*), *F-box protein 38* (*Fbxo38*), and *Dipeptidyl peptidase 8* (*Dpp8*). These extensive high-throughput data from IMPC mice provide a promising opportunity to explore genetics causing metabolic heart disease with an important translational approach.

## Introduction

Cardiovascular disease (CVD) is a world-leading health problem and encompasses a broad spectrum of disorders, including diseases of the blood vessels, the heart muscle, the electrical conduction system, and congenital heart disease. Hereditary DNA sequence variants play a role in the transmission of disease risk in almost all of them (Basson et al. 1997; Garg et al. 2005; Geisterfer-Lowrance et al. 1990; Kathiresan and Srivastava 2012; Lehrman et al. 1985; Schott et al. 1998; Wang et al. 1995). With the entire genomes of numerous species published (Hotaling et al. 2021; Jackson et al. 2021; Rhie et al. 2021; Samaha et al. 2021), the desired but as yet unmet goal is now to identify DNA sequence variants responsible for trait variation in patients (Klasberg et al. 2016; Oprea 2019).

Animal models have many facets that mimic various disease conditions in humans representing the importance for its tremendous use in biomedical research (Mukherjee et al. 2022). Small animal models have improved our understanding of the various aspects and etiologies of heart disease and provided new treatment strategies (Riehle and Bauersachs 2019; Zaragoza et al. 2011). Mice and rats are one of the most commonly used experimental animal models as they share a high degree of homology to the human genome with −20,000 protein-coding genes each (Bryda 2013). Yet, the mouse shares a similar developmental trajectory to the human heart and a comparable four-chamber morphology that greatly enhances its translational value as a model organism, despite the substantial differences in size (Krishnan et al. 2014).

The International Mouse Phenotyping Consortium, IMPC, has set the goal of systematically “knocking out” each protein-coding genes in the mouse and phenotyping these so-called knockout (KO) mice using highly standardized high-throughput tests (Brown and Moore 2012). Causal impact of a full-gene deletion will be investigated in all organ systems by comparing phenotypic characteristics of KO-mice with wild-type control mice of the same genetic C57BL/6N background (Munoz-Fuentes et al. 2018). With the focus on translatability from mouse to human disease (Cacheiro et al. 2019), the IMPC has made extensive discoveries of new links of so far poorly studied genes to disease areas, such as deafness (Bowl and Brown 2018), metabolic disorders (Rozman et al. 2018), and bone mineral density (Swan et al. 2020). In the heart, genes associated with congenital monogenic CVD (Cacheiro et al. 2023; Spielmann et al. 2022) and a heart-brain axis have recently been described (Garrett et al. 2022). These studies highlight the grand potential of KO-models from the IMPC to study the genetic influence and complementary pleiotropic roles of genes in heart disease. Freely available for the worldwide research community (https://www.mousephenotype.org/about-impc) (Koscielny et al. 2014), the IMPC is offering a largely unexplored genetic landscape that may be instrumental toward improving clinical diagnosis and management and ultimately optimizing disease prevention, early diagnosis, and treatment of heart disease.

In this review, we present the practical and methodological high-throughput applications of in vivo IMPC cardiac diagnostics, such as electrocardiography and transthoracic echocardiography. In addition, we provide insight into current advances in cardiac and metabolic phenotyping in the IMPC by describing data from benchmark examples of known disease genes, and moreover, highlight previously under-reported genes such as *Rnf10* that causes pathological changes in metabolism and cardiac function when knocked out in mice.

## Phenotyping in the International Mouse Phenotyping Consortium (IMPC)

The IMPC is a global effort with 21 phenotyping centers located on five continents. To provide robust high-quality data, the IMPC uses a series of standardized protocols as described in IMPReSS (International Mouse Phenotyping Resource of Standardized Screens https://www.mousephenotype.org/impress/index). This allows the generation of a data resource that is comparable and shareable. More so, these mouse data enable ontological annotations and thus interspecies comparisons. For each gene, a minimum of 14 homozygous KO-mice (7 females, 7 males) are phenotyped, while reference baseline levels are monitored by continuously tested wild-type controls on the same C57BL/6N background using the same protocols. In non-viable homozygous mice, heterozygous mice are tested instead. The so-called “Early Adult Pipeline” is covering a wide range of *in vivo* phenotyping procedures from 9 to 15-weeks postnatal with additional weekly body weight monitored from 4 to 16 weeks. High-level information is available for all organ systems at https://www.mousephenotype.org/understand/data-collections/.

Electrocardiography (ECG) and transthoracic echocardiography (TTE) are conducted at week 12 and detailed in the following paragraph, while metabolic data are measured at weeks 11, 13, and 14 but not described in depth in the recent study (Rozman et al. 2018).

## Cardiovascular phenotyping in the IMPC

This review is based on data from data release (DR) 17 published on July 19, 2022 at https://www.mousephenotype.org/data/previous-releases/17.0 with a total number of 8267 phenotyped genes, respectively, 8916 mutant lines in IMPC. Among those, 5457 knockout mouse lines have ECG and 2030 TTE data.

### Electrocardiography

Electrocardiography (ECG) records electrical potentials from the body surface that arise from ion flows in the heart during a cardiac cycle. Individual features in the ECG signal, peaks and troughs reflect important phases of excitation in the pacemaker cells, conduction of action potentials, and depolarization and repolarization of cardiomyocytes. The ideal placement of electrodes (leads) would be that changes in the electromagnetic field around the heart related to physiologically relevant current flows during the heart cycle are detected. One of the standard configuration of leads in clinical practice is to attach electrodes to the hands and the left leg, known as Lead II Configuration. ECG has become an established diagnostic tool and is considered the gold standard for diagnosing arrhythmias and conduction disorders, as well as for distinguishing acute coronary syndromes with ST-segment elevation from those without (Harskamp 2019). In addition to assessing symptomatic patients, ECG is also commonly used for screening purposes in primary care to timely detect silent atrial fibrillation (AF) in asymptomatic patients and/or detection of ECG abnormalities associated with coronary artery disease and a significant health burden (Hornick and Costantini 2019).

The same method is used in mice and yields a typical P-QRS-T waveform that is in principle directly comparable to the morphology of the human ECG, except for the J wave (Calvet and Seebeck 2023). This is a generally recognized feature of the mouse ECG but was omitted from this IMPC study. More specifically, there are minor differences in the nature and pattern of potassium channel activation during the development of an action potential in mice and humans (Boukens et al. 2014); these are particularly important to note for specific ion channel activity, such as in studies of channelopathies-induced arrhythmias. For such specific forms of arrhythmia, it is recommended to use rabbit and guinea pig models beyond the mouse with a combination of ECG data within these mammalian models to obtain the most meaningful data (Joukar 2021).

**Fig. 1:**
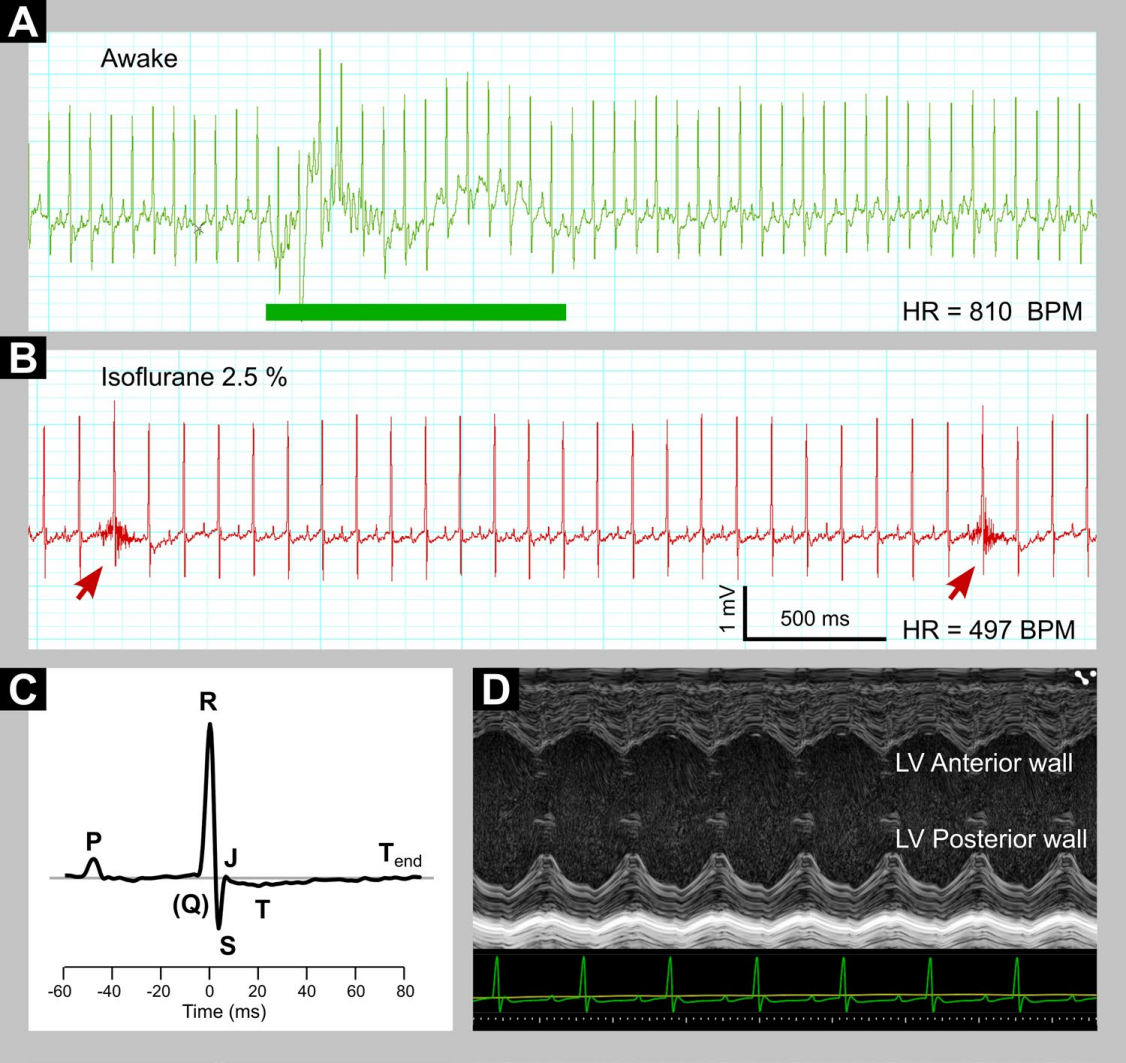
Overview of different mouse ECGs. **A** Recording of conscious ECG. Green rectangle denotes a portion of signal with artifacts caused by animal movements. **B** ECG in Lead II configuration under isoflurane anesthesia is at the same scale as in A. Arrows indicate breath artifacts. **C** P-QRS-T complex of an anesthetized mouse, an average of 20 subsequent beats aligned at R peak. Note missing Q wave and negatively oriented wave T. **D** ECG monitoring at time of anesthetized sonography of the heart (e.g. echocardiography). Left ventricle (LV) was imaged in short axis M-mode to visualize the relation of anterior and posterior wall movement to ECG waves. Note: For a discussion of anesthetized versus non-anesthetized ECG please refer to the respective section on the main text

Electrodes detect electrical events; however, ECG does not reflect the heart contraction per se. To obtain more complex information about in vivo heart function, it is useful to combine ECG with heart imaging by ultrasonography (Fig. 1) or to use telemetric systems (Calvet and Seebeck 2023).

The interpretation of ECG in mice is based on the same quantitative parameters as in humans. The IMPC high-throughput phenotyping requires capturing and analysis of the following mandatory parameters: Heart rate (HR), heart rate variability (HRV), RR interval duration (RR) and coefficient of variance (CV) of RR, PQ interval duration (PQ), PR interval duration (PR), QRS interval duration (QRS), QT interval duration (QT), and its value corrected for heart rate (QTc) based on the Mitchell formula (Mitchell et al. 1998), and QT / QTc dispersion.

### High-throughput electrocardiography recording in mice

**Fig. 2:**
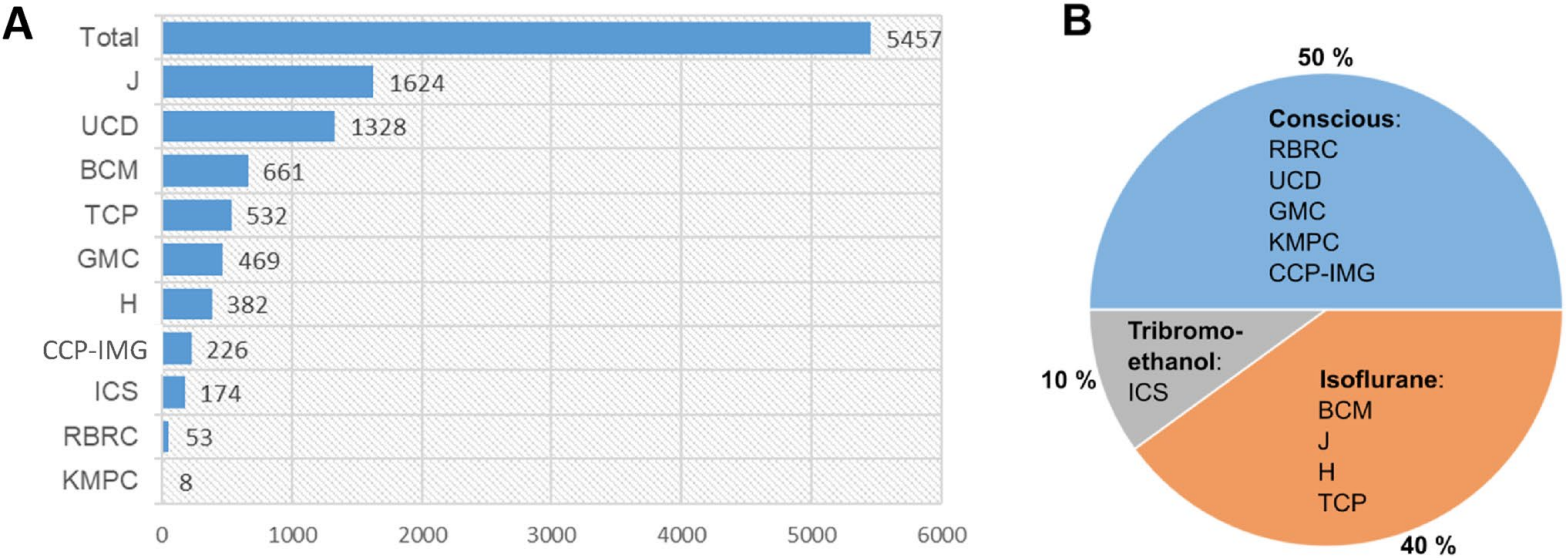
Overview of DR17 collection. **A** Total of 5457 knockout lines with ECG data classified by IMPC centers worldwide; data is presented in numbers of knockout mouse lines. Abbreviations for centers: Jackson Laboratory, USA (J); University of California, USA (UCD), Baylor College of Medicine, USA (BMC); Centre for Phenogenomics, Canada (TCP), German Mouse Clinic, Germany (GMC); Medical Research Council, Mary Lyon Center, Harwell, United Kingdom (H), Czech Centre for Phenogenomics, Institut of Molecular Genetics, Czech Republic (CCP-IMG), Institut Clinique de la Souris, France (ICS), RIKEN BioResource Research Center, Japan (RBRC) and Korean Mouse Phenotyping Center, Korea (KMPC)). **B** ECG data split by optional administration of anesthesia (%): conscious, isoflurane or tribromoethanol anesthesia

Out of a grand total of 8916 KO-mouse lines, ECG data have been collected in 5457 lines (61.4%) of young adults at 12 weeks of age by 10 IMPC centers globally. ECG data are collected in mice without (conscious) or under anesthesia. The latter comprises, depending on the IMPC centers, isoflurane inhalation and tribromoethanol intraperitoneal anesthesia. Figure 2A shows the distribution of ECG data across the contributing centers and Fig. 2B presents the ECG data split by consciousness and anesthesia with the majority of data (50%) collected in conscious, whereas 40% in mice under isoflurane and 10% under tribromoethanol anesthesia.

#### Recording procedure

Most IMPC centers perform non-invasive ECG recordings in conscious mice, followed by ECGs under isoflurane inhalation or, for a slightly smaller proportion of mice, intraperitoneal tribromoethanol anesthesia. In conscious ECG, several conditions are essential to avoid elevated stress levels in the mice. The laboratory should be calm and illuminated with dim light. While recording, no other activities should be carried out. Animals are transported to the laboratory 30 min prior to recording for acclimation. The cardiovascular system is highly temporally organized (Wager-Smith and Kay 2000) and exhibits pronounced circadian fluctuations, so measurements on the mice should always be performed at the same time as described in the IMPReSS protocols (https://www.mousephenotype.org/impress).

**Fig. 3:**
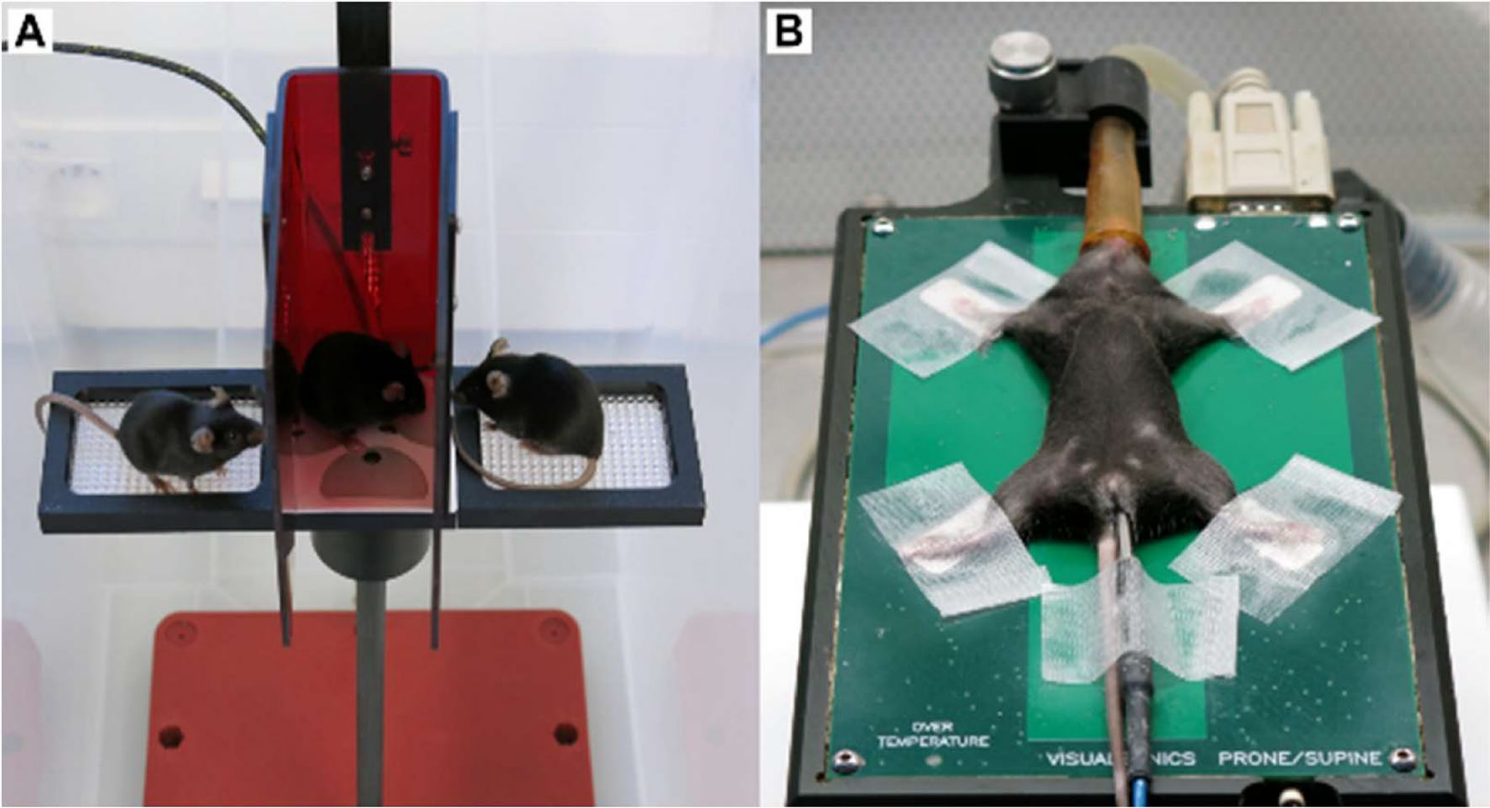
**A** ECGenie platform for recording a conscious ECG with the mouse in the red-squared center sitting on paw (touch sensitive) electrodes. **B** Visual Sonic recording platform for ECG recording under anesthesia; mouse is fixed with tape on a preheated plate, rectal probe and nose in cone; two positions are possible like belly-down or belly-up; here we show the latter possibility that allows simultaneous transthoracic imaging of the heart by ultrasonography

While recording, the mouse is standing on an elevated ECGenie platform (Mouse Specifics, Inc.) with three conducting plate electrodes on the floor and surrounded with red plexiglas to create a comfortable small compartment (Fig. 3A) whereas pen platforms proved to be more stressful for a mouse (https://mousespecifics.com/). Our experience showed that placing the ECG system inside a large transparent plastic box is minimizing the risk of mouse escape. Many IMPC centers use two ECG platforms in parallel to increase the throughput. Prior to each experiment, a new set of electrodes is applied and after each recording, a base disinfected with 70% ethanol is essential to clean the platform. The ECGenie platform consists of three standing positions for mice. The center position is surrounded by a red plexiglas box where the recorded mouse is placed. The other two positions to the left and right side of the plexiglas are used for placing cage mates to minimize the stress in mice.

#### ECG signal detection

Once the mouse and the platform are ready for recording, the heartbeat signal is digitized by an AD convertor with a 2-kHz sampling frequency and recorded by LabChart software set to an amplitude range of ± 5 mV and band-pass filtering between 3 and 100 Hz, with a 50-Hz notch filter on. In a high-throughput setting, the recording length is typically between 5 and 10 min, with 5 min being the minimum duration. During this recording phase, the animal should be calm and ideally in a stable resting position with paws on at least 2/3 electrodes.

#### Data analysis

Raw data recording consists of periods of clean ECG signal interleaved with noisy parts due to moving artifacts of conscious mice. Preferentially, rather noise-free signals are taken for analysis. Mostly, these are the recordings that are at the end of the recording session. This data is considered to be representative of a “resting state.” As an IMPC guideline, sections of a ‘clean’ ECG without motion and noise artifacts must contain at least 60 heartbeats in total. Data are saved separately for each mouse.

#### ECGenie software

The recorded data from LabChart software are exported into ECGenie software (Mouse Specifics, Inc.). ECGenie detects basic features in the clean signal and provides numeric data on the ECG peaks and intervals. This data is averaged. As an alternative, ECG data analysis can be done by various other commercially available programs, such as LabChart (ADInstruments), AcqKnowledge (BIOPAC), LabVIEW (National Instruments), Ponemah (Data Sciences International), or LabScribe (iWorks Systems, Inc.).

### Electrocardiography recording in anesthetized mice

Two types of anesthesia are performed in the IMPC. One is isoflurane short-term inhalation anesthesia and to a lesser extent tribromoethanol intraperitoneal anesthesia. Here, we describe the standard operating procedure (SOP) for isoflurane ECG recordings. ECG SOPs in conscious state (Spielmann et al. 2022) and under tribromoethanol anesthesia have been previously described (Meyer and Fish 2005).

#### Recording

Induction concentration should be around 5% of isoflurane in oxygen at a flow rate of 1 l/min. Once sedation is confirmed in the animal, it should be quickly transferred to a heated recording platform (Physiological Unit, VisualSonics) with four-plate electrodes. ECG lead plates are covered with ECG gel and all 4 paws of the mouse lying on its back are taped to electrodes by tape. A rectal thermometer is used to monitor and record the core body temperature. Eyes are protected from drying by designated mouse eye cream (Fig. 3B). Isoflurane concentration is now reduced to 2–3% during recording.

#### ECG signal detection

Anesthetized ECG is obtained in lead II configuration, band-pass filtering is set to 3–100 Hz, and the signal is digitized at 2 kHz of sampling rate. ECG signal is recorded by LabChart software. This configuration allows keeping the seamless ECG recording on for short- or long-time recordings.

### Pros and cons of anesthetized and conscious ECG

A conscious ECG in mice offers a highly translational approach without influencing the autonomic regulatory systems. ECG in conscious mice provides a highly translational approach for the cardiac monitoring that does not influence the autonomic regulatory system, unlike anesthesia and is similar to standard ECG monitoring in humans. Thus, ECG parameters obtained from conscious mice can be directly transferred to humans and corresponding cardiac abnormalities. A conscious ECG is a gentle approach for monitoring the mouse heart, as the handling time from cage to the ECGenie platform is short with a minimized animal burden, however, it is impossible to determine the exact lead configuration. The animals keep changing their posture throughout the recording and find different resting poses with paws in varying positions and moving artifacts can impair the recorded signal. The signal-to-noise ratio is higher in single measurements as produced for a high-throughput setting but can be improved by repeated measurements to reach full adaptation to the ECG settings for each mouse. However, this is not feasible in a high-throughput pipeline. Moreover, ECG parameters such as heart rate variability have a limited frequency range when calculated from recordings as short as several seconds (i.e., tens to hundreds of beats).

Anesthetized mice allow recording an ECG signal that is substantially cleaner than in the conscious state. A sedated mouse offers tighter and more stable contact of the limbs to electrodes which is even more improved by applying gel for optimized signal conductivity. An anesthetized mouse does not move, and this allows the recording of ECG free from movement artifacts. Under anesthesia, the ECG signal can be supplemented with other physiological data, such as respiration rate and body temperature. In addition to ECG, the mouse is well set on the same platform for a sonography of the heart. However, there are substantial limitations when administering anesthesia*.* Isoflurane is the predominantly used inhalational anesthetic in short-term experimentation with mice partly because of its moderate cardio-depressive effects in comparison to those of the injectable agents (Constantinides et al. 2011; Tomsits et al. 2023). The advantage of stable measurement under isoflurane inhalation anesthesia is the orchestrated moderate decline in HR and respiration. The HR during anesthesia declines to about 420–480 beats per minute (BPM) compared to normal values of about 730–800 BPM when conscious. Furthermore, during an anesthetized ECG procedure, thermoregulation is vital for maintenance of homeostasis in mice (Janssen and Smits 2002). In summary, for a representative ECG, the concentration of isoflurane as well as the body temperature of the mouse must be controlled, which requires more effort than conscious ECG.

### Transthoracic echocardiography (TTE)

Echocardiography provides a rapid non-invasive assessment of the heart, including evaluation of myocardial thickness, function, valvular disease, pericardial pathology, and chamber size. M-mode imaging, two-dimensional analysis, and Doppler echocardiography can also provide information on blood velocity, cardiac pressure gradients, and valve areas (O’Riordan et al. 2023; Steeds et al. 2017).

In the IMPC, high-throughput transthoracic echocardiography (TTE) represents a useful, non-invasive method to visualize cardiovascular structures in mice and evaluate cardiac function in real time. Apart from the obvious size differences, the mouse and human heart are anatomically similar throughout development (Wessels and Sedmera 2003). There are differentiations in orientation, however, as the mouse heart is more vertically oriented than the human heart. Nevertheless, the parasternal views with long axis (PLAX) and short axis (PSAX) can be easily obtained. Yet, the orientation of the heart and the small size of the chest cause the acquisition of the apical view to be difficult and poorly reproducible, thereby preventing a complete analysis of diastolic function most particularly of the right ventricle. Due to its planar position, small size, and complex shape, the right ventricle is not well visualized on transthoracic echocardiography and is consequently often neglected in mouse diagnostics (Scherrer-Crosbie and Thibault 2008; Zacchigna et al. 2021). Mice have a 10 times higher heart rate than humans and very small cardiovascular dimensions. These species differences require special small laboratory animal devices for high-frequency ultrasound as provided by Vevo Imaging Systems (Visual Sonics, Fuji Films). The IMPC uses TTE data to assess primarily the dimensions of left ventricle (LV) in end-distolic and end-systolic, LV wall thickness, and LV pumping function, whereas the right ventricle is omitted in high-throughput. LV-based heart diagnostics allows us to study causalities between phenotype <—> genotype and to explore how single-gene depletion affects the heart (Cacheiro et al. 2023; Spielmann et al. 2022).

### High-throughput transthoracic echocardiography (TTE) imaging in mice

**Fig. 4:**
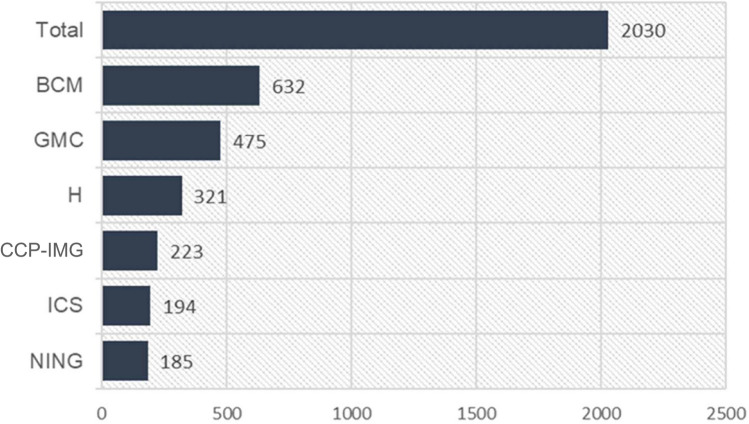
Overview of transthoracic echocardiography data in the IMPC split by centers and presented as numbers of KO-mouse lines

In DR 17, the set of TTE data currently represents 2030 single-gene KO-mouse lines from early adult (12-week-old) mice (Fig. 4). In most mutant lines (76.6%), TTE is performed under anesthesia, whereas in 23.4% of the lines data are collected in conscious mice (GMC) in the early adult pipeline. Of those using anesthesia, 87.5% is performed under isoflurane (BCM, H, CCP-IMG, and NING) and 12.5% under tribromoethanol (ICS).

#### Isoflurane anesthesia preparation

Exemplary for all centers using isoflurane anesthesia, we describe how it is performed at the Czech Center for Phenogenomics (CCP-IMG). For most parts, this procedure is identical to the isoflurane anesthesia described in the ECG section above.

In brief, the initial isoflurane anesthesia is performed in the induction chamber containing a mixture of 4–5% isoflurane (flow 1 l/min) followed by inhalation anesthesia through the nasal mask with a lower 1–3% mixture of oxygen and isoflurane resulting in a flow of 0.3–0.5 l/min. The mouse is placed on a pre-warmed stage (38 °C) to avoid hypothermia. Fur is removed using a shaving gel (commercially available human shaving gel, Veet Minima). After two to five minutes, the shaving gel is removed by a wet tissue. The pre-warmed and water-based sonographic gel (lubricating gel) is applied on the shaved area with a piece of cotton or a cotton swab. The stage with the fixed mouse must be adjusted to the special positioning and the ultrasound probe. At CCP-IMG, a MS400 (alternatively MS550) transducer is used to start the TTE procedure. Overall, the whole procedure including the start of anesthesia, shaving the chest, and accommodating the mouse to the platform takes between 5 and 10 min.

#### TTE equipment used in IMPC

VisualSonics equipment (FujiFilm, Vevo 770, 2100 or 3100 platform) is used by 92% of all IMPC centers, whereas one center, ICS, uses Philips equipment (Sonos 5500). Over the last ten IMPC years, different platforms were used starting with Vevo770 and expanding to Vevo3100 following upgrades and technical advances. In the early adult pipeline, the Vevo2100 is used in five of six IMPC centers (BCM, CCP-IMG, H, ICS, and NING), whereas one center (GMC) is using the Vevo3100 platform; these technical distinctions, however, have no effect on the overall IMPC data.

#### High-throughput TTE recording in IMPC mice

**Fig. 5:**
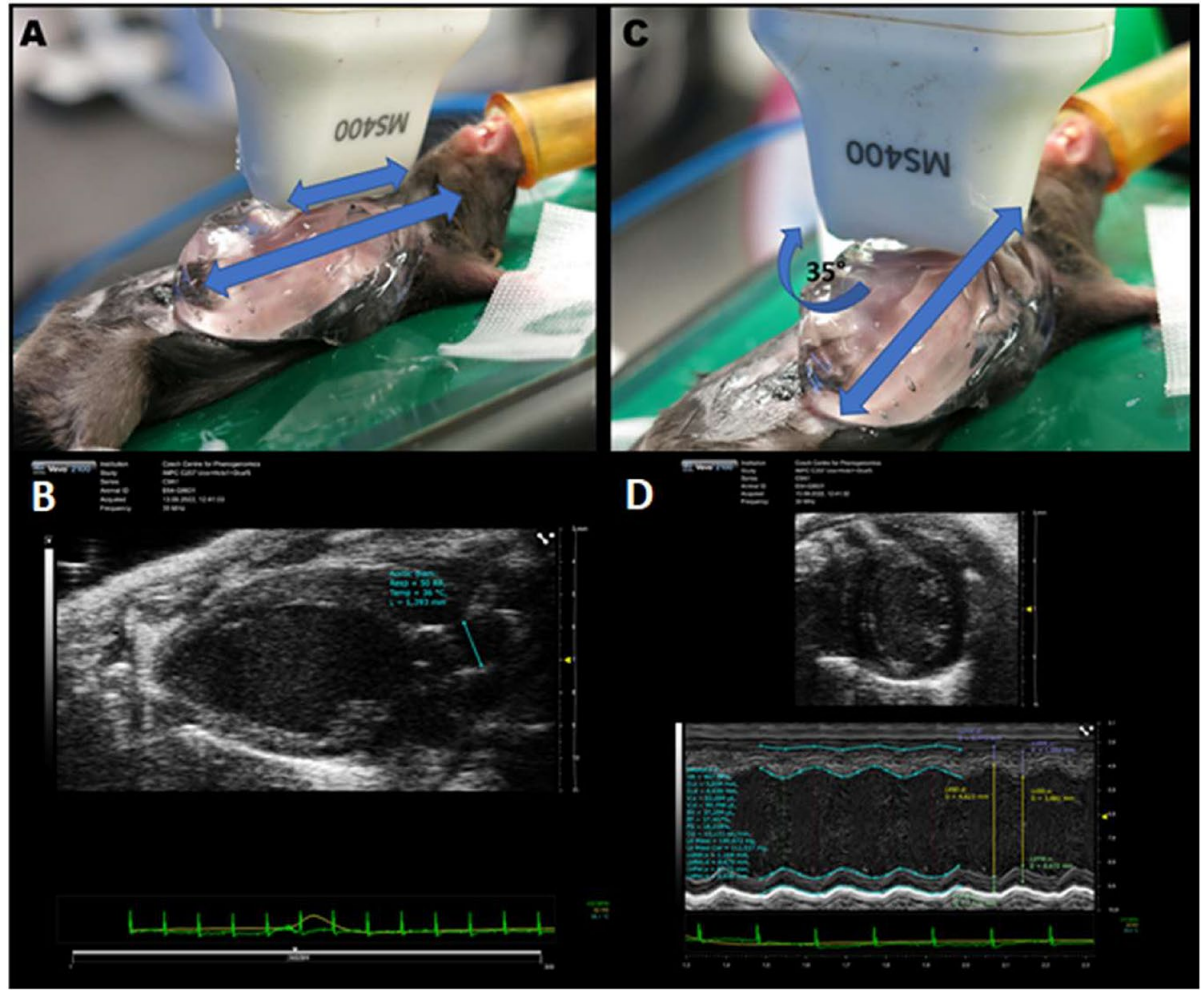
Transthoracic echocardiography in mice. The mouse is placed on the pre-heated platform, the paws of the mouse are fixed with tape, the chest of the mouse is shaved and ultrasound gel is applied. ECG and body temperature are monitored. **A** Position of the transducer (short blue arrow) during parasternal long axis recording, when the probe is oriented in the longitudinal direction of the long body axis (long blue arrow). Image of ultrasound record with ECG signal (in the bottom). **B** The PLAX is used for aorta diameter analysis (blue line). Position of the probe during parasternal short axis recording. **C** The probe is turned approximately 35° clockwise to a longitudinally oriented probe. **D** By rotating the transducer approximately 90° clockwise, parasternal short axis is obtained with an ultrasound image and AutoLV analysis. The papillary muscles are in one line (at the level of yellow arrows)

Despite the distinctions in the status of the mice (i.e., conscious versus anesthetized), the procedures follow harmonized SOPs (see Impress: https://www.mousephenotype.org/impress/ProcedureInfo?action=list&procID=654) for TTE measurements and performed identically by all IMPC centers. Parasternal long-axis (PLAX) view is the starting point for high-throughput TTE diagnostics in mice where the transducer is positioned in a vertical direction with its notch pointing toward the animal’s head (Fig. 5A). Then, the transducer is rotated in 35° counterclockwise. The visible anatomy of the mouse heart at the landmarks of aorta and apex should also contain the LV anterior and posterior wall. In B-mode, an image of LV in PLAX is recorded. Besides LV morphology, the long-axis image is also used (but not mandatory) for measurement of aortic diameter in the IMPC (Fig. 5B). This aortic measurement is done by drawing a line, which is perpendicular to the wall of the aorta, and software automatically provides the value of aortic diameter. In advanced recording, but not mandatory for the IMPC high throughput, an M-mode of PLAX can be obtained and used for functional analysis.

Next, the parasternal short-axis (PSAX) view is obtained by rotating the transducer approximately 90° clockwise as PSAX represents the plane perpendicular to the longitudinal axis of the heart (Fig. 5C). Following the 90° rotation, the Y-axis may need further adjustment to obtain the proper view. PSAX measurement uses a short-axis view of the heart aligning the base of the heart and the apex. PSAX gives a cross-sectional view of the LV with the required visible anatomy of the LV and papillary muscles as a landmark. Usually both, B-mode and M-mode images are recorded. For M-mode imaging, the axis should be placed in the center of the LV with the papillary muscles clearly visible aligned with the guideline provided by the system (Fig. 5D). Based on a two-dimensional M-mode LV image, multiple parameters are calculated by the system using the Teichholz formula (Teichholz et al. 1976) with high-throughput IMPC diagnostics being based on:

Ejection fraction, fractional shortening, cardiac output, stroke volume, LV anterior wall thickness in end-diastole and end-systole (LVAWd, LVAWs), LV posterior wall thickness in end-diastole and end-systole (LVPWd, LVPWs), and LV internal diameter in end-diastole and end-systole (LVIDd, LVIDs).

At the end of an imaging session, ultrasound gel is removed with water-dampened gauze and the mouse placed back in the cage where it is monitored post-examination (up to 1 h).

#### Standard analysis of high-throughput TTE in mice

In the IMPC, mandatory TTE data are generated in the M-Mode capturing end-systolic and end-diastolic dimensions and functional derivates such as LVAWd and LVAWs, LVPWd and LVPWs, LVIDd and LVIDs, and aortic diameter (Dao); ejection fraction (EF), fractional shortening (FS), cardiac output (CO), and stroke volume (SV); and heart rate (HR) with respiration rate (RR) and for anesthetized mice the body temperature via rectal node.

High-throughput imaging generates large data sets. As of yet, TTE data cannot be fully evaluated manually. Typically, only two consecutive heartbeats are annotated in a single echocardiogram, based on the American Society of Echocardiography recommendations (Wiegers et al. 2019). Some centers are using the semi-automatic VevoLab software (AutoLV) generated for anesthetized mouse TTE analysis, whereas such an automatic solution is missing for conscious TTE data. AutoLV is applied to PSAX imaging of the heart, starting at the end of the diastolic cycle, from which the software generates the automatic measurement. AutoLV can be edited in case of ‘a human disagreement with the software countering’ of the TTE signal waves. Manual annotation has been previously described in detail (Moreth et al. 2014). With respect to AutoLV and manual annotation, these distinctions in TTE evaluation, however, have no effect on the overall IMPC data.

### Pros and cons of anesthetized and conscious TTE

TTE in conscious mice is performed very rarely and requires a lot of training and high level of mouse handling expertise from the operator. The German Mouse Clinic (GMC) is the only group in the IMPC that has been performing this method at a high expert level for many years (Moreth et al. 2014). Due to the fixation of the operator in the neck grip and the very short examination time, the burden on the awake mouse during TTE is minimal. Conscious TTE offers a highly translational approach as it does not affect the autonomic control systems by anesthesia just like in the patient. Thus, TTE parameters obtained in mice can be directly translated to humans and corresponding morphological and functional cardiac abnormalities. Application of anesthesia, on the other hand, allows TTE diagnostics under stable conditions and simplified circumstances for the operator. Thus, the transducer is held in a fixation and can be minimally adjusted for optimal image quality, whereas conscious TTE requires free-hand transduction (transducer in one hand-mouse in the other hand). Motion artifacts and poor/unusable images are therefore very rare in anesthetized measurements yet with the influence of the anesthetic on cardiac function and TTE data.

#### TTE quality control in the IMPC

All data collected by the IMPC passes strict quality control filters implemented by dedicated individuals in the team. High-level details on IMPC quality control measures and filters can be accessed under https://www.mousephenotype.org/phenodcc/qc-documentation/. This data is then statistically analyzed to identify significant phenotypes associated with the KO-mouse line at a significance level of 10^–4^. The IMPC uses a toolkit OpenStats (previously PhenStat, both R packages), which helps to apply appropriate statistical methods for each data type and for the identification of abnormal phenotypes with an emphasis on high-throughput dataflow. If the mutant genotype effect represents a significant change from the control group at the IMPC level of 0.0001 for continuous and categorical measurements, the IMPC pipeline will associate a Mammalian Phenotype (MP) term to the data (Mammalian Phenotype Ontology). Next, data integration methods are employed to assess the translational relevance of each mutant line toward human biology (https://www.mousephenotype.org/help/data-analysis/). In general, the IMPC has adopted a significance level of 10^–4^ (*P* < 0.0001), unless otherwise indicated, to maintain a false discovery rate (FDR) below 5%.

The website of IMPC procedures also provides a simple explanation of the effect of individual parameters on the heart with its ontology term https://www.mousephenotype.org/impress/OntologyInfo?action=list&procID=654#28681.

### Large-scale cardiovascular phenotyping in the IMPC

In DR17 (DR17, https://www.mousephenotype.org/data/release), 8916 early adult mouse lines were phenotyped and made publicly available with 59.4% (5295/8916) having a cardiovascular system phenotype, whereas 3621 lines had a heart phenotype. Split by zygosity, 2600 lines showed phenotypes in homozygous mice, 983 in heterozygous mice, whereas 38 were observed in hemizygous mice. Among those, 437 phenotype associations were found for ECG and 317 for TTE in young adult mice.

A comprehensive list of genotype <  > phenotype associations can be accessed here:https://www.mousephenotype.org/data/previous-releases/17.0.

#### Effect of age on the heart

Aging in mice is increasingly being studied, but differences in study design and background make it difficult to draw general conclusions about aging in the mouse heart (Xie et al. 2022; Zhang et al. 2021). To investigate the effects of aging on the heart, the IMPC has established a “late adult pipeline” where selected lines of interest (e.g., based on early adult data or request from external specialist in the field) are nominated for aging of at least 52 weeks.

**Fig. 6:**
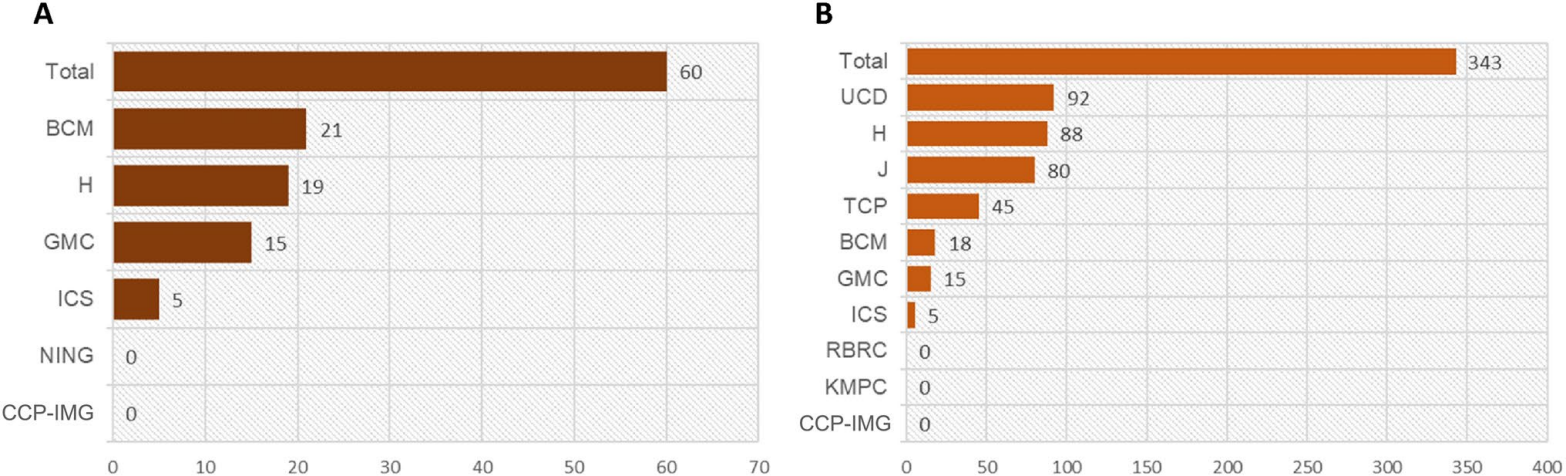
Overview of data collection in late adult (LA) mice. **A** Overview of KO-mouse lines phenotyped in LA classified by centers for TTE. **B** KO-mouse lines per center with ECG data in LA mice

The phenotyping of aged mice is more challenging because KO-mice may not be viable until the age of 52 weeks and are more susceptible to age-related burden. Up to date (DR17), a total of 60 KO-mouse lines have been phenotyped in the late adult (LA) pipeline. Data were released mostly by Baylor College of Medicine, USA (BCM, 40.4%), Helmholtz Center Munich, Germany (GMC, 25%), and 9.6% by Institute Clinique de la Souris, France ICS (Fig. 6A). In this pipeline, only Visual Sonic equipment is used. BCM, GMC, and ICS use the model Vevo2100 and Vevo3100, whereas MRC Harwell works with Vevo770. TTE recordings are obtained under isoflurane anesthesia at BCM, MRC Harwell, and ICS or in the conscious state at GMC.

A total of 322 KO-mouse lines have been phenotyped with ECG data in the LA pipeline. Data have been released mostly by UC Davis, USA (UCD, 28%), Jaxson, USA (J, 25%), MRC Harwell (H, 21%), Toronto Center of Phenotyping (TCP, 14%), Baylor College of Medicine, USA (BCM, 6%), German Mouse Clinic (GMC, 5%), and 2% by Institut Clinique de la Souris, France ICS (Fig. 6B). LA data are presented on the IMPC portal using a LA heat map where all mouse lines for which the IMPC has collected aging data up to now are included. This heat map is interactive allowing searching for a gene of interest or a procedure by its name, i.e., ECG (https://www.mousephenotype.org/understand/data-collections/late-adult-data/).

### Large-scale metabolic phenotyping in the IMPC

In EA mice, homeostasis and/or metabolic system-related phenotypes were highly present with 76.6% (6832/8916) of the KO-mouse lines having a homeostasis and/or metabolic system-related phenotype, wherein 2084 lines had no homeostasis and/or metabolic phenotype at all. Split by zygosity, 5013 KO-mouse lines showed metabolic phenotypes in homozygous mice, 1721 in heterozygous mice, whereas 98 were observed in hemizygous mice. Among those, 2965 phenotype associations were found for body composition, 4345 for clinical chemistry parameters, 70 blood-level insulin phenotypes, and 780 phenotype <  > genotype associations for intraperitoneal glucose tolerance. Importantly, the absolute number of lines may differ depending on the parameter set. A comprehensive list of genotype <  > phenotype associations can be accessed here:https://www.mousephenotype.org/data/previous-releases/17.0.

### Genes providing a link between cardiovascular and metabolic phenotypes in mice

**Fig. 7:**
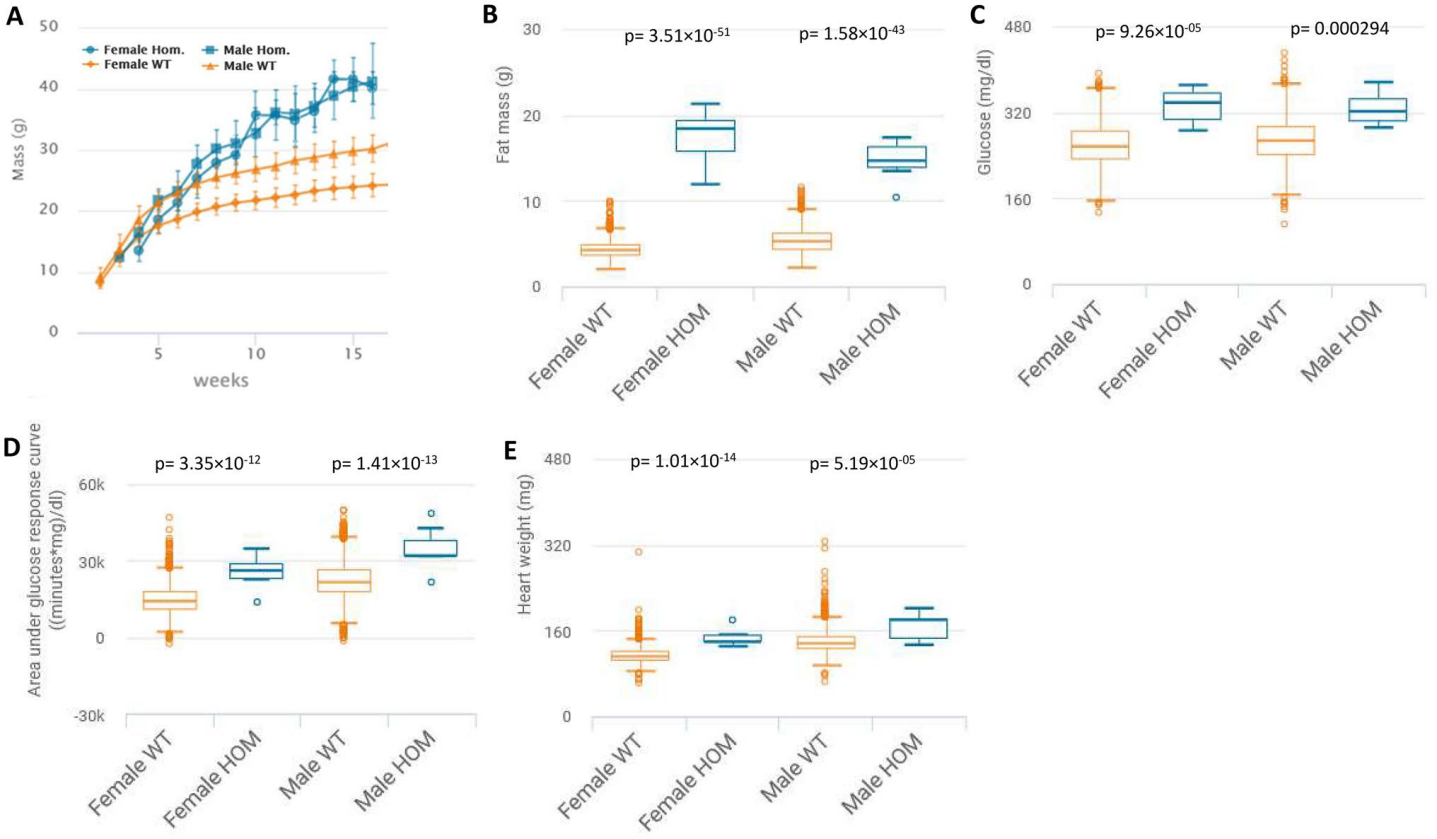
Representative metabolic and cardiovascular phenotypes in the *Bbs5*-KO *mouse*. **A** *Bbs5*-KO mice show massively increased body weight (g) over time evident in male and female KO-mice when compared to controls. The charts show the results of measuring body weight curve in 7 *Bbs5*-KO female, 7 male *Bbs5*-KO mutants compared to 3520 female, 3515 male controls. **B** *Bbs5*-KO mice have enormously increased total body fat amount (g) from a body composition (DEXA lean/fat) assay compared to controls with a genotype*female p-value = 3.51 × 10^−51^ and genotype*male p-value = 1.58 × 10^−43^. The chart shows the results of measuring fat mass in 7 *Bbs5*-KO female, 7 male *Bbs5*-KO mutants compared to 1881 female, 1864 male controls. Statistics: Linear Mixed Model framework, LME, including weight with a phenotype threshold value of 1e-04 for both sexes equally. **C** *Bbs5*-KO mice have significantly increased glucose (mg/dl) from a clinical chemistry phenotypic assay compared to controls with a genotype*female p-value = 9.26 × 10^−05^ and genotype*male p-value = 0.000294. The chart shows the results of measuring fat mass in 7 *Bbs5*-KO female, 7 male *Bbs5*-KO mutants compared to 2209 female, 2194 male controls. Statistics: Linear Mixed Model framework, LME, including weight with a phenotype threshold value of 1e-04 for both sexes equally. **D** *Bbs5*-KO mice have significantly increased area under glucose response curve (minutes*mg/dl) from intraperitoneal glucose tolerance test (IPGTT) phenotypic assay compared to controls with a genotype*female p-value = 3.35 × 10^−12^ and genotype*male p-value = 1.41 × 10^−13^. The chart shows the results of measuring lean/body weight in 7 *Bbs5*-KO female, 7 male *Bbs5*-KO mutants compared to 2678 female, 2673 male controls. Statistics: Linear Mixed Model framework, LME, including weight with a phenotype threshold value of 1e-04 for both sexes equally. **E** *Bbs5*-KO mice have significantly increased heart weight from an organ weight phenotypic assay compared to controls with a genotype*female p-value = 1.01 × 10^−14^ and genotype*male p-value = 5.19 × 10^−5^. The chart shows the results of measuring of measuring heart weight in 6 *Bbs5*-KO female, 7 male *Bbs5*-KO mutants compared to 2253 female, 2219 male controls. Statistics: Linear Mixed Model framework, LME, including weight with a phenotype threshold value of 1e-04 for both sexes equally. High-level detail for this KO-mouse line can be accessed here: https://www.mousephenotype.org/data/genes/MGI:1919819

In the IMPC, metabolic and cardiovascular diseases represent important research priorities to assist in the identification of genes that cause congenital heart defects, obesity, diabetes, and the metabolic syndrome in humans. Alterations in energy metabolism are found in a large number of rare and common human diseases of genetic or environmental origin (Rossignol 2015). Impairments in energy metabolism have negative effects on the heart and contribute to the severity of heart failure, as the contraction and relaxation of the heart is disturbed. This heart failure is caused by abnormalities in metabolic pathways that lead to reduced energy production, energy transfer and energy use. These impairments affect the periphery by leading to early muscular fatigue and exercise intolerance (Lopaschuk et al. 2021). Using mouse models to provide transformative understanding of the genetic basis of multi-systemic diseases like metabolically induced heart failure is thus one of the key IMPC missions. Genes that have already been thoroughly investigated are neglected in the majority of cases, which is why many known genes are significantly underrepresented in the IMPC (White et al. 2013); nonetheless, we introduce herein KO-mouse models for some recognized metabolic and cardiovascular genes, such as the *leptin receptor* (*Lepr*), *leptin* (*Lep*), and *Bardet–Biedl syndrome 5* (*Bbs5*). All these genes, when depleted in the mouse, show metabolic and cardiovascular alterations of comparable nature to those observed in humans (Katsiki et al. 2018) (Pomeroy et al. 2021) (Cacheiro et al. 2023). Consistent with studies in pediatric patients carrying *BBS5* mutations (Pomeroy et al. 2021), substantial young age weight gains were observed in *Bbs5*-KO-mice (Fig. 7A). A battery of very specific metabolic abnormalities like significantly increased fat mass but lower lean mass, hyperglycemia after food deprivation, and impaired glucose tolerance together with increased heart weights were evident in *Bbs5*-KO-mice compared to C57BL/6N controls (Fig. 7B–E). The IMPC provides high-level details for *Lepr*, *Lep*, and *Bbs5* when using these links:

- *Lepr:* https://www.mousephenotype.org/data/genes/MGI:104993;
- *Lep:* https://www.mousephenotype.org/data/genes/MGI:104663;
- Bbs5*:* https://www.mousephenotype.org/data/genes/MGI:1919819

**Fig. 8:**
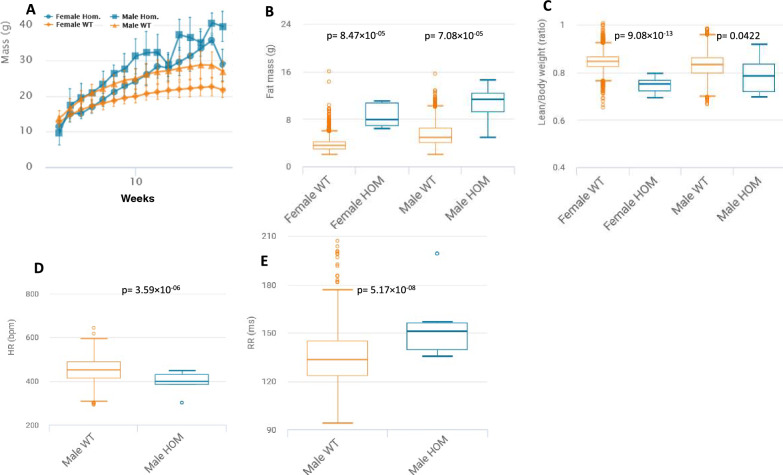
Representative metabolic and cardiovascular phenotypes in the *Dpp8*-KO mouse. **A** *Dpp8*-KO mice show increased body weight over time evident in male and female KO-mice when compared to controls. The charts show the results of measuring body weight curve in 8 *Dpp8*-KO female, 7 male *Dpp8*-KO mutants compared to 3677 female, 3685 male controls. **B** *Dpp8*-KO mice have significantly increased total body fat amount from a body composition (DEXA lean/fat) assay compared to controls with a genotype*female p-value = 8.47 × 10^−05^ and genotype*male p-value = 7.08 × 10^−05^. The chart shows the results of measuring fat mass in 8 *Dpp8*-KO female, 7 male *Dpp8*-KO mutants compared to 2626 female, 2568 male controls. Statistics: Linear Mixed Model framework, LME, including weight with a phenotype threshold value of 1e-04 for both sexes equally. **C** *Dpp8*-KO mice have significantly decreased lean/body weight from a body composition (DEXA lean/fat) assay compared to controls with a genotype*female p-value = 9.08 × 10^−13^ and genotype*male p-value = 0.0422. The chart shows the results of measuring lean/body weight in 8 *Dpp8*-KO female, 7 male *Dpp8*-KO mutants compared to 2615 female, 2559 male controls. Statistics: Linear Mixed Model framework, LME, including weight with a phenotype threshold value of 1e-04 for both sexes equally. **D** *Dpp8*-KO mice have significantly decreased heart rate from an electrocardiogram recording compared to controls with a genotype*male p-value = 3.59 × 10^−06^. The chart shows the results of measuring of measuring heart rate in 7 male *Dpp8*-KO mutants compared to 736 male controls (females not shown). Statistics: Linear Mixed Model framework, LME, including weight with a phenotype threshold value of 1e-04. **E** *Dpp8*-KO mice show significantly prolonged RR intervals from an electrocardiogram recording compared to controls with a genotype*male p-value = 5.17 × 10^−08^. The chart shows the results of measuring of measuring RR intervals in 7 male *Dpp8*-KO mutants compared to 736 male controls (females not shown). Statistics: Linear Mixed Model framework, LME, including weight with a phenotype threshold value of 1e-04. High-level detail for this KO-mouse line can be accessed here: https://www.mousephenotype.org/data/genes/MGI:1921638

More so, the IMPC provides evidence for the utility of yet unassociated loss-of-function candidate genes for diseases affecting the metabolic and cardiovascular systems. IMPC data translated from basic research allow prioritization of inherited but undiagnosed mutations in patients suffering from heart diseases caused by disturbances in metabolism and thus identify a human variant as causative. Genes such as the *RING finger protein 10* (*Rfn10*), *F-box protein 38* (*Fbxo38*), and *Dipeptidyl peptidase 8* (*Dpp8*) exemplify this group. In mice, *Rnf10* depletion caused a large panel of alterations in metabolically relevant traits in presence of heart impairments (Rozman et al. 2018). In humans, *RNF10* has been identified as a member of a new RING finger protein class containing a C3HC4-type zinc finger motif (Seki et al. 2000) possibly linked to neuronal differentiation, viral infections and cancer (Dinamarca et al. 2016; Hoshikawa et al. 2008; Malik et al. 2013; Mateu-Huertas et al. 2014; Rodrigues-Lisoni et al. 2010). In Pima Indians, however, a population characterized with high-risk for diet-induced obesity and its sequelae, a strong metabolic association, was found of single-nucleotide polymorphisms in *RNF10* with adiposity and type 2 diabetes mellitus (T2DM) (Huang et al. 2014). This is highlighting an example of how the IMPC effort offers potential disease models for the study of mechanistic underpinnings in specific patient subpopulations. *Fbxo38*, a gene associated to distal hereditary motor neuronopathy type IID (OMIM: 615,575) causes, when depleted in mice, a set of hematopoietic alterations, decreased body weight and growth with increased heart rate in mice. *Dpp8,* a gene with no human disease association, causes at depletion significantly increased total body fat amount with decreased lean body mass content at reduced heart rate with concurrently prolonged RR intervals (Fig. 8A–E). These are two examples among many regarding the way the IMPC is contributing new data to metabolic induced disease models and delineating hitherto unrecognized genotypes <  > phenotypes. The IMPC provides high-level details for *Rfn10*, *Fbxo38*, and *Dpp8* when using these links:

- *Rfn10:* https://www.mousephenotype.org/data/genes/MGI:1859162
- *Fbxo38:* https://www.mousephenotype.org/data/genes/MGI:2444639
- *Dpp8:* https://www.mousephenotype.org/data/genes/MGI:1921638

## Conclusions and outlook

The IMPC has been working with KO-mouse models for a decade, providing large-scale data across all organ systems to the public. This rich data resource has provided new insights into previously unassociated genes in various diseases. In the area of heart disease, the IMPC has made great progress targeting “variants of unknown significance” causally classified (Spielmann et al. 2022) or common cardiac genes captured by KO-mice (Cacheiro et al. 2023). Herein, we demonstrate the diagnostic prerequisites for the consortium’s cardiac screening and the promising opportunity to explore genes that cause metabolically induced heart disease.

High-throughput data from mice have their limitations in understanding disease mechanisms and genomic effects on the expressivity of disease phenotypes. Thus, in the same genotype, different expressions of the same phenotype can be triggered by the extent of expressivity (Dickinson et al. 2016). This distinction of a trait expression is challenging both in the mouse model and patients (Kathiresan and Srivastava 2012). Further, pleiotropy, penetrance, and non-genetic factors confer that even when a single gene is disrupted, the genotype does not “equate" to a particular phenotype. This complexity has multiple consequences and is therefore imperative to conduct research in a collaborative global effort involving different mammalian and fish models together with patient data. To this end, the IMPC promotes active collaboration with clinicians and researchers from around the world with the overarching goal of exploring the genetic basis of disease using highly standardized mouse models to provide an important initial guide. Anticipating that this will then ultimately underpin the prevention, detection, diagnosis and treatment of various heart diseases, including metabolic heart disease (https://www.mousephenotype.org/about-impc/collaborations/). If you are interested in collaborating, please contact info@mousephenotype.org.

## Data Availability

IMPC data and information are publically available: https://www.mousephenotype.org/; DR17 is accessible here: https://www.mousephenotype.org/data/previous-releases/17.0.
